# The association between employment status and health among British adults with and without intellectual impairments: cross-sectional analyses of a cohort study

**DOI:** 10.1186/s12889-018-5337-5

**Published:** 2018-03-27

**Authors:** Eric Emerson, Chris Hatton, Susannah Baines, Janet Robertson

**Affiliations:** 1 Centre for Disability Research, Lancaster University, Lancaster, LA1 4YT UK; 2 Centre for Disability Research and Policy, Faculty of Health Sciences, University of Sydney, Camperdown, NSW 2006 Australia

**Keywords:** Employment, Health, Intellectual disability, Intellectual impairment, Borderline intellectual functioning, Cognitive ability

## Abstract

**Background:**

There exists a well established link between employment status and health, with unemployment being associated with poorer health. Much less is known about the association between economic inactivity and health, especially among people with disabilities. Our aim is to determine whether the association between employment status and health is similar for adults with and adults without intellectual impairment.

**Methods:**

Using nationally representative data from the 1970 British Cohort Study, we undertook a series of cross sectional analyses of the association between employment status and health (self-reported general health, mental health) among British adults with and without intellectual impairments at ages 26, 30, 34, 38 and 42.

**Results:**

People with intellectual disability and borderline intellectual functioning had markedly lower employment rates and poorer health than other participants at all waves of data collection. When compared with participants in full-time employment the prevalence of poorer self rated health and mental health was higher among participants with and without intellectual impairment who were in either part-time employment or were economically inactive at all ages. When compared with participants in employment the prevalence of poorer self rated health and mental health was higher among participants with and without intellectual impairment who were in the economically inactive categories of unemployment, education/training and ill/disabled at all ages. Intellectual disability status appeared to moderate the strength of the relationship between economic activity and self-rated health and, to a much lesser extent, the relationship between economic activity and mental health. In all instances the moderation indicated a stronger association among participants without intellectual impairment.

**Conclusions:**

The results provide substantive evidence to suggest that the nature of the well-established association between employment and better health is similar for British adults with and without intellectual impairments. The results do, however, indicate that the magnitude of the effect involved differed. Further research is needed to identify mechanisms that may underlie this difference.

## Background

There exists a well established link between employment status and health, with unemployment being associated with poorer health [1–4]. This association appears to be accounted for by two distinct processes; health selection (healthier people are more likely to gain and retain employment), and specific health benefits associated with employment [1, 3, 5–7]. The latter is considered of sufficient importance that ensuring equality of access to non-exploitative employment is commonly considered a key policy option for reducing health inequities [8, 9].

The vast majority of the literature on the association between employment and health is based on analyses of people who are considered to be part of the current labour force (those employed or actively seeking employment). Much less attention has been paid to the health of people who are ‘economically inactive’ or ‘workless’ (i.e., people who are not working for a variety of reasons, including studying or providing care, or are deemed ‘unfit’ for work) [10, 11]. This is somewhat paradoxical as in many high income countries the economically inactive population is significantly greater than the unemployed population. In the UK, for example, from January to March 2017 in the 16 to 64 age group there were 8.83 million people (21.5%) who were economically inactive, compared to just 1.54 million unemployed people (3.7%) [12]. The economically inactive group was comprised of: 2.30 million people who were not looking for work because they were studying; 2.21 million people (of which 1.95 million were women) who were not looking for work because they were looking after the family or home; 1.99 million people who were not looking for work because of poor health/disability; and 1.17 million people who were not looking for work because they were retired.

The available research that does exist on the health of the economically inactive suggests that: (1) the health of economically inactive adults is broadly similar to that of the unemployed; [10, 13–16] (2) moving from economic inactivity due to sickness or disability into employment is associated with a decreased risk of reporting poor health; [15, 17–21] and (3) both unemployment and economic inactivity are highly significant predictors of the onset of limiting longstanding illness [18].

Policies that seek to reduce health inequity need to take account of the situation of groups who are particularly vulnerable to exposure to well established social determinants of poor health or who may be particularly vulnerable to the effects of such exposures [9, 22]. People with disabilities are one such group [23]. While it is clear that people with disabilities have significantly reduced access to employment, [24, 25] little is known about the association between employment status and health for people with disabilities [19, 26]. Results from the sparse literature that does exist suggests: (1) people with disabilities who were unemployed or economically inactive have poorer mental health when compared with people with disabilities who were employed; [26] and (2) people with poor health or disabilities are much more vulnerable to job loss and less likely to enter paid employment than those with good health [27].

Disability, however, is associated with a wide range of health conditions or impairments. Increasing evidence suggests that some impairments are associated with greater levels of disadvantage. For example, intellectual impairments have been associated with much lower rates of employment than those experienced by people with disabilities generally [28, 29].

In this paper we investigate the association between employment status and health among two groups of people with intellectual impairments; people with intellectual disability and people with borderline intellectual functioning. Intellectual disability refers to a significant general impairment in intellectual functioning that is acquired during childhood. It is commonly defined as scoring more than two standard deviations below the population mean on tests of general intelligence (IQ < 70). While empirical estimates of the prevalence of intellectual disability vary widely, [30] it has been estimated that approximately 2% of the adult population of England have an intellectual disability [29]. Borderline intellectual functioning is most commonly defined as scoring between one and two standard deviations below the population mean on tests of general intelligence (IQ 70–84), with an estimated prevalence of 12–15% of the adult population [31, 32]. We are aware of only two population-based studies that have examined the association between employment status and health among people with intellectual impairments. First, in a nationally representative survey of 1273 English adults with mild/moderate intellectual disability employment rates were reported to be 26% and employment was significantly associated with more positive self-rated health, especially among women [33]. Second, in a nationally representative UK sample of adults in the age range 16–49, fulltime employment rates (16 h or more per week) were reported to be 15% for 279 adults with intellectual disability and 58% for 2297 adults without intellectual disability. In multivariate analyses full time employment was associated with more positive self-rated health for adults with and without intellectual disability [34].

Given the dearth of existing studies in this area, the aim of the present paper is to determine whether the association between employment status and health is similar for adults with and adults without intellectual impairment.

## Methods

We undertook secondary analysis of data from eight waves of the 1970 British Cohort Study (BCS70). Details of BCS70 are available in two cohort profiles [35, 36] and in an extensive series of technical reports and supporting documentation (e.g., interview questionnaires) that are available for download from the UK Data Service (https://www.ukdataservice.ac.uk/). Key methodological aspects of the study are briefly summarized below.

BCS70 is a nationally representative individual level survey following up over 17,000 children born during 1 week in the UK in 1970. In the first wave of data collection (soon after birth) information was collected from midwives on 17,198 infants (the cohort members). Since then, information has been collected on various aspects of the lives of cohort members at age 5 (*n* = 12,939), 10 (*n* = 14,350), 16 (*n* = 11,206), 26 (*n* = 8654), 30 (*n* = 10,833), 34 (*n* = 9316), 38 (*n* = 8874) and 42 (*n* = 9717) [37–39]. The surveys cover a wide range of issues such as: health; health behaviors; wellbeing; educational attainment; employment and occupation; financial status; social and civic participation; social support; family formation and crime. Data collection in adulthood has been by postal survey (age 26) and computer aided interviews with study members (ages 30, 34, 38, and 42). At age 38 the interview was conducted via telephone. At all other ages the interviews were conducted face-to-face. Information on proxy responding is only available at ages 30, 34 and 42. Proxy responses for participants with borderline intellectual functioning was 0.3% at age 30, 0.2% at age 34 and 0.1% at age 42. Proxy responses for participants with intellectual disability was 8.7% at age 30, 9.2% at age 34 and 9.7% at age 42. BCS70 is currently managed by the Centre for Longitudinal Studies at University College London (http://www.cls.ioe.ac.uk/) and is funded by the UK’s Economic and Social Research Council (http://www.esrc.ac.uk/). Confidentialised data from the age 5, 10, 16, 26, 30, 34, 38 and 42 follow-up surveys were downloaded from the UK Data Service [40–47].

### Identifying participants with intellectual impairments

While BCS70 included direct measurements of child cognitive functioning at ages 5, 10 and 16, [48] at no age were complete validated tests of IQ administered. Instead, a range of brief tests were administered, some drawn from validated tests of IQ, others assessing attainment that is likely to be related to IQ. The tests were administered by Health Visitors at age 5 and the child’s teacher at age 10. On both occasions written guidelines were provided regarding test administration. In similar circumstances a number of previous studies have used factor analytic procedures to establish the presence of a general cognitive ability factor across tests (traditionally named ‘*g’*) and, if present and accounting for an acceptable proportion of common variance, have used standardized scores on the first extracted component (*g*) as a proxy for IQ [48–51].

We followed this practice by deriving a proxy measure of IQ from the results of age 10 cognitive testing and, if these were not available, age 5 cognitive testing. This decision was based on three considerations: (1) cognitive testing at age 10 included four subscales of a well validated test of IQ, the British Ability Scale; [52, 53] (2) the cognitive tests administered at age 10 had greater internal consistency than those administered at age 5 (alpha = 0.89 vs 0.58); and (3) cognitive test results at age 10 were available for a significantly greater percentage of children than at age 16 (87% vs 52%).

At age 10, eight tests were administered: the Shortened Edinburgh Reading Test; [54] the Friendly Maths Test; [48] the Pictorial Language Comprehension Test; [48] the Spelling Dictation task; [48] and four subscales of the British Ability Scales, Word Definitions, Word Similarities, Recall of Digits and Matrices [52]. In total, 12,885 (87%) of all children participating in the age 10 survey completed at least one assessment and 11,134 (75%) children completed all eight assessments [48]. In order to maximize use of participants’ data and to reduce potential bias resulting from exclusion of partial non-respondents (those who completed at least one, but not all tests), missing data for partial respondents were imputed using multiple imputation routines in IBM SPSS 22. Five parallel data sets were imputed for each partial non-respondent and then averaged to create the final imputed data. Principal components analysis was used to establish the presence of a general cognitive ability factor across tests and standardized scores on the first component were extracted as a proxy indicator for IQ [48–51]. At age 10, the first extracted component accounted for 59% of the variance of initial eigenvalues with all tests loading positively on the component (loading range 0.55 for BAS Digit Recall to 0.88 for the Shortened Edinburgh Reading Test).

Age 5 cognitive test results were available for an additional 2568 children for who no age 10 cognitive test data were available. At age five, five tests were administered: the Copying Designs Test; [55] the English Picture Vocabulary Test; [56] the Human Figure Drawing (Draw-a-Person) Test; [57] the Complete a Profile Test; [58] and the Schonell Reading Test [59]. In total, 13,059 (99%) of all children participating in the age 5 survey completed at least one assessment, with 11,254 (86%) children completing all five assessments [48]. We followed the procedures outlined above to: (1) impute partially missing cognitive test results; (2) establish the presence of a general cognitive ability factor across tests (*g*); (3) use standardized scores on *g* as an indicator of IQ at age 5. At age 5, the first extracted component accounted for 41% of the variance of initial eigenvalues with all tests loading positively on the component (loading range 0.47–0.76).

This procedure generated a proxy measure of IQ for 15,453 participants. Of these, 426 (2.8%) were functioning in the IQ range associated with intellectual disability (IQ 70 or below), 2108 (13.6%) were functioning in the borderline intellectual functioning range (IQ range 71–85) [31] and 12,919 (83.6%) were functioning in a higher IQ range (IQ 86+).

### Health indicators

#### Self-reported general health

A single-item measure of self-reported general health was collected at each adult wave. However, question formats and response options varied across waves. At age 26 the postal questionnaire asked ‘*How would you describe your general health?*’ with four response options (‘*excellent/good/ fair/ poor*’). At age 30 cohort members were asked ‘*How would you describe your health generally? Would you say it is ..*’ followed by the same four response options used at age 26. At age 34 cohort members were asked ‘*Please think back over the last 12 months about how your health has been. Compared to people of your own age, would you say that your health has on the whole been ..*’ followed by five response options (‘*excellent/good/ fair/ poor/very poor*’). At ages 38 and 42 cohort members were asked ‘*In general, would you say your health is...*’ followed by five response options (‘*excellent/very good/good/ fair/ poor*’). Given the variation in questions and response options over time we derived a simple binary measure of health (excellent/very good/good vs. fair/poor/very poor).

#### Mental health

At ages 26 and 30 the 24-item *Malaise Inventory* was used to measure levels of psychological distress or depression [55]. The potential presence of a mental health problem was identified by a score or eight or more [60]. At ages 34 and 42 an abbreviated 9-item version of the *Malaise Inventory* was used, with potential mental health problem being identified by a score or four or more.

### Economic activity

A derived measure summarizing the current economic activity of cohort members is included in the published dataset. The 12-category classification of economic activity, based on that used by the UK’s Office for National Statistics, includes: full-time employment (working 30+ hours per week); part-time employment (working less than 30 h per week); full-time self-employment; part-time self-employment; unemployment (actively looking for and available for work); full-time education; participating in a Government scheme for employment training; temporary sickness/disability; permanent sickness/disability; looking after the home/family; retirement; other. From these data we derived two variables of economic activity; overall economic activity and detailed economic activity. *Overall economic activity* had three categories: employed full-time (as employee or self-employed); employed part-time (as employee or self-employed); economically inactive (all other categories). *Detailed economic activity* had six categories: employed (as employee or self-employed, full or part-time); unemployed; education/training (full-time education or participating in a Government scheme for employment training); ill/disabled (temporary or permanent); at home; other (including retirement).

### Approach to analysis

Initial exploratory analyses indicated significantly higher attrition rates among cohort members with intellectual impairments when compared to cohort members without intellectual impairments (Table 1). We addressed the issue of bias due to attrition by imputing all missing data (arising from either wave or item non-response) as previous analyses of BCS70 had indicated that well specified imputation models were preferable to the use of sample weights [61]. Imputation was undertaken using the multiple imputation routines in SPSS 22 to create five parallel data sets. Predictor variables for the imputation models were selected on the basis of known association with attrition and data availability [62, 63]. The final variables included were: cohort member gender and ethnicity; intellectual impairment status (see above); health indicators at all ages (see above); indicators of family socio-economic position and child health at ages 5, 10 and 16; self-assessed financial position, economic activity, social class and de facto marital status at ages 26, 30, 34, 38 and 42; disability, basic skill problems and obesity at ages 26, 29, 34 and 42; educational attainment at ages 26, 29, 34 and 38; sense of control at ages 26, 34 and 42; life satisfaction at ages 29, 34 and 42; housing tenure at ages 29, 34 and 38; emotional support at ages 29 and 34; and evidence of hearing or vision problems or epilepsy at any age. All results reported in the results section were based on analysis of pooled data.

**Table 1:** Non-participation rates in BCS70 from age 5

	Age 10	Age 16	Age 26	Age 30	Age 34	Age 38	Age 42
ID	7.0%	38.5%	68.8%	47.2%	61.0%	71.8%	59.6%
BIF	6.5%	37.0%	59.9%	41.4%	53.8%	61.7%	52.4%
Others	6.4%	27.0%	41.8%	29.0%	37.9%	42.0%	37.2%

Note: *ID* intellectual disability, *BIF* borderline intellectual functioning

Initial exploratory analysis also indicated that intellectual impairments were more common among males (intellectual disability 3.0% vs. 2.5%; borderline intellectual functioning 14.3% vs. 12.9%) and minority ethnic groups (intellectual disability 8.2% vs. 2.4%; borderline intellectual functioning 26.0% vs. 12.9%). Given these between group differences on participant characteristics that are also potentially related to health, all analyses (unless specified) were adjusted to take account of the potential confounding impact of gender and ethnicity. We chose not to control for other potentially ‘confounding’ variables associated with educational attainment or living situation as these are likely to be determined (in part) by intellectual functioning and, often more importantly, societal responses to people with impaired intellectual functioning.

In the first stage of analysis we describe the employment status of adults with and without intellectual impairments at each wave. In the second stage of analysis we used Poisson regression with robust standard errors to estimate the association between economic activity status and health outcomes at each age stratified by intellectual impairment status. In addition we repeated the analyses on the full sample of participants including intellectual impairment status and the two way interaction between intellectual impairment status and employment status as variables. In the third stage of analysis we used Poisson regression with robust standard errors to estimate the association between the number of times exposed to economic inactivity within the dataset and health indicators at age 42. In the fourth stage of the analysis we used Poisson regression with robust standard errors to estimate the association between a finer grained categorization of economic activity status and health outcomes at each age again stratified by intellectual impairment status (those with either intellectual disability or borderline intellectual functioning and other participants). The relatively small sample size for participants with intellectual disability precluded undertaking these analyses on them as a distinct group. Again, we repeated these analyses on the full sample of participants including intellectual impairment status and the two way interaction between intellectual impairment status and employment status as variables. All analyses were undertaken in IBM SPSS 24.

## Results

Unadjusted percentage employment and health status are presented in Table 2. Adjusted prevalence rate ratios (PRRs) estimating the association between economic activity status and health outcomes at each age separately for the three groups of participants are presented in Table 3. Prevalence of poor health was greater in every analysis across the two health indicators, age and participant groups for economically inactive participants and participants in part-time employment when compared to that of participants in full-time employment. In 51 of the 54 comparisons this difference was statistically significant. . Across these analyses the median PRR was highest for participants without impaired intellectual functioning (others 3.32, borderline intellectual functioning 2.62, intellectual disability 2.78). Tests for interactions between intellectual impairment status and economic activity revealed few significant differences. With regard to self-rated health, at age 26 there was a significant interaction between borderline intellectual functioning and part-time employment, at age 34 there was a significant interaction between intellectual disability and economic inactivity, and at age 42 there was a significant interaction between borderline intellectual functioning and economic inactivity. In all instances the effect sizes for the intellectually impaired groups were significantly lower than expected. There were no significant interactions between intellectual impairment status and economic activity with regard to mental health outcomes.

**Table 2:** Unadjusted employment and health status by age and intellectual impairment status

Age	Intellectual impairment status	Employment status			Health status	
		Full time employed	Part time employed	Unemployed or economically inactive	‘Poor’ or ‘fair’ self-rated health	Potential mental health problem
26	ID	33.7%	12.7%	54.7%	49.1%	40.7%
	BIF	45.1%	10.1%	44.8%	40.3%	32.9%
	Others	62.1%	8.2%	29.7%	28.2%	21.5%
30	ID	38.1%	22.1%	39.8%	37.4%	35.8%
	BIF	47.4%	21.7%	30.9%	33.2%	28.6%
	Others	61.5%	18.0%	20.5%	22.1%	19.3%
34	ID	38.3%	34.9%	39.8%	62.4%	33.4%
	BIF	43.6%	33.0%	23.4%	55.5%	27.6%
	Others	55.2%	27.9%	16.9%	40.3%	20.4%
38	ID	36.6%	29.9%	33.6%	40.0%	n/a
	BIF	42.6%	28.6%	28.9%	35.1%	n/a
	Others	53.9%	25.8%	20.4%	24.4%	n/a
42	ID	36.0%	19.4%	44.6%	40.6%	44.8%
	BIF	46.3%	18.8%	34.9%	33.9%	35.6%
	Others	58.4%	19.7%	21.9%	22.2%	26.3%

Notes: *ID* intellectual disability, *BIF* borderline intellectual functioning

**Table 3:** Adjusted^a^ prevalence rate ratios (with 95% confidence intervals) for poorer health associated with economic activity status for adults with intellectual disability, adults with borderline intellectual impairment and other adults

	ID			BIF			Others		
	FT	PT	EI	FT	PT	EI	FT	PT	EI
‘Poor’ or ‘fair’ self-rated health									
Age 26	1.00	4.14 (2.44–7.02)	3.66 (2.14–6.25)	1.00	3.41* (2.87–4.06)	3.03 (2.54–3.63)	1.00	4.40 (4.11–4.70)	3.36 (3.12–3.62)
Age 30	1.00	3.27 (1.95–5.48)	3.09 (1.87–5.10)	1.00	2.64 (2.18–3.19)	2.92 (2.43–3.49)	1.00	3.01 (2.76–3.27)	3.19 (2.94–3.47)
Age 34	1.00	2.94 (2.11–4.10)	2.63* (1.88–3.68)	1.00	3.48 (2.98–4.06)	3.63 (3.12–4.23)	1.00	3.89 (3.66–4.13)	4.31 (4.06–4.57)
Age 38	1.00	4.74 (2.00–11.22)	5.42 (2.32–12.70)	1.00	4.44 (3.29–6.00)	5.37 (4.00–7.21)	1.00	4.18 (3.76–4.64)	5.72 (5.17–6.33)
Age 42	1.00	1.81 (0.92–3.55)	3.09 (1.71–5.57)	1.00	1.85 (1.44–2.37)	2.60* (2.11–3.19)	1.00	2.08 (1.85–2.34)	3.50 (3.20–3.84)
Potential mental health problem									
Age 26	1.00	1.88 (1.15–3.09)	1.96 (1.19–3.23)	1.00	2.20 (1.81–2.68)	2.26 (1.85–2.77)	1.00	2.33 (2.25–2.53)	2.46 (2.25–2.70)
Age 30	1.00	2.26 (1.24–4.13)	3.63 (2.10–6.28)	1.00	2.52 (2.01–3.17)	2.77 (2.23–3.45)	1.00	3.15 (2.86–3.47)	3.37 (3.07–3.71)
Age 34	1.00	1.36 (0.79–2.33)	1.45 (0.87–2.43)	1.00	1.28 (1.00–1.65)	1.58 (1.26–1.98)	1.00	1.35 (1.21–1.50)	1.70 (1.54–1.87)
Age 42	1.00	1.77 (1.02–3.08)	2.63 (1.60–4.31)	1.00	1.88 (1.49–2.36)	2.51 (2.07–3.05)	1.00	1.65 (1.50–1.81)	2.57 (2.38–2.78)

Notes: * significant interaction

^a^adjusted for gender and ethnicity

*ID* intellectual disability, *BIF* borderline intellectual functioning, *FT* full-time employment (reference), *PT* part-time employment; EI = economically inactive

**Fig. 1:**
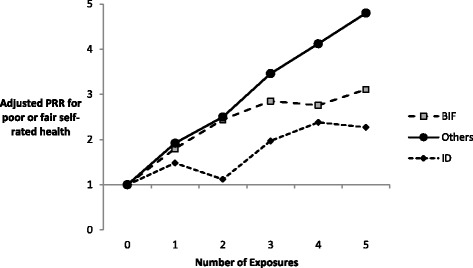
Adjusted prevalence rate ratios for ‘poor’ or ‘fair’ self-rated health associated with number of exposures to economic inactivity for adults with intellectual disability, adults with borderline intellectual impairment and other adults

**Fig. 2:**
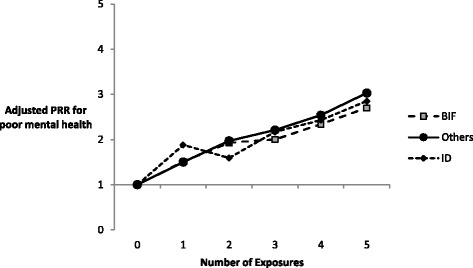
Adjusted prevalence rate ratios for poorer mental health associated with number of exposures to economic inactivity for adults with intellectual disability, adults with borderline intellectual impairment and other adults

PRRs estimating the association between the number of times exposed to economic inactivity and health outcomes at age 42 are presented in Figs. 1 and 2 separately for the three groups of participants. For all three groups the prevalence of poor health increased with number of exposures to economic inactivity. The effect was stronger for self-rated health and, for self-rated health only, for participants without intellectual impairments.

PRRs estimating the association between detailed economic activity status and health outcomes at each age are presented in Table 4 separately for the two groups of participants (with and without intellectual impairment). The prevalence of poor health was greater in all analyses comparing employment (the reference group) with the three ecomonically inactive categories of unemployment, education/training and ill/disabled. All but one of these 54 comparisons was statistically significant. . Across these analyses the median PRR was lower for participants with intellectual impairment (others 3.67, intellectual impairment 2.64). Tests for interactions between intellectual impairment status and economic activity revealed a number of significant differences with regard to self-rated health, but few for mental health. With regard to self-rated health: (1) at all ages there were significant interactions between intellectual impairment and the two economically inactive categories of unemployment and education training; (2) at ages 26, 34, 38 and 42 there were significant interactions between intellectual impairment and the economically inactive category of ill/disabled; and (3) at age 30 there was a significant interaction between intellectual impairment and the economically inactive category of ‘at home’. With regard to mental health: (1) at age 30 there was a significant interaction between intellectual impairment and the economically inactive category of unemployed; (2) at ages 30 and 34 there were significant interactions between intellectual impairment and the economically inactive category of education/training; and (3) at ages 26 and 30 there were significant interactions between intellectual impairment and the economically inactive category of ill/disabled. Again, in all instances the effect sizes for the intellectually impaired groups were significantly lower than expected.

**Table 4:** Adjusted^a^ prevalence rate ratios (with 95% confidence intervals) for poorer health associated with detailed economic activity status for adults with and without intellectual impairments

		Employed	Unemployed	Education/Training	Ill/Disabled	At Home	Other
‘Poor’ or ‘fair’ self-rated health							
Age 26	ID/BIF	1.00	3.30 (2.84–3.84)*	3.52 (3.03–4.09)*	3.80 (3.22–4.48)*	1.09 (0.77–1.56)	1.46 (0.63–3.39)
	Others	1.00	4.79 (4.48–5.12)	4.71 (4.39–5.05)	5.31 (4.86–5.79)	0.92 (0.72–1.16)	0.68 (0.39–1.20)
Age 30	ID/BIF	1.00	2.67 (2.28–3.13)*	2.85 (2.45–3.32)*	3.09 (2.61–3.65)	1.85 (1.48–2.31)*	0.94 (0.34–2.65)
	Others	1.00	3.21 (2.95–3.49)	3.73 (3.45–4.03)	3.77 (3.40–4.17)	1.47 (1.25–1.74)	1.67 (1.14–2.46)
Age 34	ID/BIF	1.00	3.18 (2.84–3.56)*	3.38 (3.03–3.76)*	3.33 (2.98–3.71)*	1.80 (1.53–2.12)	1.88 (1.16–3.06)
	Others	1.00	4.48 (4.25–4.72)	4.79 (4.56–5.03)	4.86 (4.62–5.10)	2.06 (1.89–2.26)	2.01 (1.59–2.53)
Age 38	ID/BIF	1.00	3.51 (2.79–4.43)*	3.83 (3.09–4.74)*	4.43 (3.58–5.48)*	3.33 (2.55–4.33)	2.43 (1.13–5.21)
	Others	1.00	4.70 (4.22–5.18)	5.49 (5.01–6.01)	6.66 (6.07–7.29)	3.08 (2.68–3.54)	2.07 (1.20–3.56)
Age 42	ID/BIF	1.00	2.41 (1.89–3.08)*	2.14 (1.77–2.59)*	2.64 (2.22–3.14)*	2.41 (1.96–2.97)	1.95 (0.87–4.40)
	Others	1.00	3.20 (2.76–3.70)	2.91 (2.63–3.22)	3.76 (3.44–4.10)	2.74 (2.43–3.09)	2.12 (1.42–3.17)
Potential mental health problem							
Age 26	ID/BIF	1.00	2.09 (1.76–2.48)	2.45 (2.07–2.89)	2.11 (1.69–2.63)*	1.29 (0.96–1.74)	0.96 (0.35–2.60)
	Others	1.00	2.44 (2.24–2.67)	2.81 (2.57–3.08)	3.31 (2.90–3.78)	1.49 (1.25–1.77)	0.97 (0.60–1.56)
Age 30	ID/BIF	1.00	2.26 (1.87–2.74)*	2.40 (2.00–2.89)*	3.40 (2.82–4.09)*	1.84 (1.43–2.36)	1.54 (0.64–3.71)
	Others	1.00	3.59 (3.26–3.94)	3.67 (3.35–4.02)	4.43 (3.97–4.94)	1.52 (1.29–1.80)	1.72 (1.14–2.61)
Age 34	ID/BIF	1.00	1.66 (1.33–2.08)	1.06 (0.85–1.33)*	1.72 (1.41–2.10)	1.31 (1.00–1.71)	1.44 (0.61–3.42)
	Others	1.00	1.43 (1.24–1.64)	1.64 (1.47–1.82)	1.95 (1.75–2.17)	1.35 (1.17–1.56)	1.17 (0.76–1.80)
Age 42	ID/BIF	1.00	1.95 (1.53–2.48)	2.23 (1.89–2.62)	2.46 (2.11–2.87)	1.94 (1.59–2.36)	1.84 (0.95–3.86)
	Others	1.00	2.17 (1.88–2.51)	2.56 (2.36–2.78)	2.70 (2.50–2.91)	2.00 (1.81–2.22)	1.56 (1.07–2.28)

Notes: * significant interaction

^a^adjusted for gender and ethnicity

*ID* intellectual disability, *BIF* borderline intellectual functioning, *FT* full-time employment (reference), *PT* part-time employment, *EI* economically inactive

## Discussion

We undertook a series of cross sectional analyses of the association between employment status and health among British adults with and without intellectual impairments. People with intellectual disability and borderline intellectual functioning had markedly lower employment rates and poorer health than other participants at all waves of data collection. Overall, the results indicate that: (1) when compared with participants in full-time employment the prevalence of poorer self rated health and mental health was higher among participants with and without intellectual impairment who were in either part-time employment or were economically inactive at all ages; (2) when compared with participants in employment the prevalence of poorer self rated health and mental health was higher among participants with and without intellectual impairment who were in the economically inactive categories of unemployment, education/training and ill/disabled at all ages; (3) intellectual disability status appeared to moderate the strength of the relationship between economic activity and self-rated health and, to a much lesser extent, the relationship between economic activity and mental health; (4) in all instances the moderation indicated a stronger association among participants without intellectual impairment.

Our results are consistent with those of the few studies that have previously examined the association between the health and employment status of people with disabilities [26, 27]. They are the first, to our knowledge, to extend this research to people with borderline intellectual functioning; a sizable population sub-group whose risk of poor health is reasonably well established [32].

We can only speculate on the possible causes of these differences in effect sizes. As noted previously, the association between employment status and health appears to be accounted for by two distinct processes; health selection (healthier people are more likely to gain and retain employment), and the specific health benefits associated with employment [1, 3, 5–7]. Differential health selection could account for these results if health status played a less prominent role in securing and retaining employment among adults with intellectual impairments. Possible mechanisms could include: (1) variation in the impact of health selection across employment sectors (e.g., health selection effects could be weaker in those sectors in which people with intellectual impairments are more likely to be employed); (2) the operation of specialist employment support programs for adults with intellectual disability [64] could potentially reduce the impact of health selection among this group. Alternatively, the results could reflect between-group differences in the benefits of employment arising, for example, from adults with intellectual impairments being more likely to be employed in less health promoting occupations. While there exists a burgeoning literature on the association between working conditions and health, [65, 66] remarkably little is known about the employment conditions experienced by people with disabilities. The limited literature does, however, indicate that, if employed, people with disabilities may be at increased risk of employment under adverse or ‘precarious’ conditions [67].

The main strength of the present study lies in its use of a population-based cohort in which it is possible to derive a proxy measure of childhood IQ. However, six limitations should be kept in mind while considering the implications of the study. First, attrition rates were significant, especially among cohort members with intellectual impairments. High attrition rates in longer term cohort studies are common, they do, however, introduce potential biases into the data. While our use of imputation to address this issue was based on previous research undertaken with this cohort, [61] we cannot rule out biases arising from factors that were either unmeasured in the surveys or that it was not possible to incorporate in the imputation models. Second, at no age were complete validated tests of IQ administered in BCS70. As a result we derived a proxy measure of IQ [48–51]. The association between our proxy measure and IQ measured by comprehensive validated IQ tests is unknown. Third, there were changes to the wording of interview questions and response options over time. As a result, evidence of variation over time in levels of health outcomes needs to be treated with caution. Fourth, while single item questions on self-rated health are commonly used in health research and have been shown to be reasonable predictors of mortality, [68, 69] we are not aware of any research which has tested the validity of these measures among people with intellectual impairments. Fifth, the use of multiple tests of interaction terms increases the risk of type 1 error. However, ore conclusion that intellectual impairment status may moderate the association between employment status and health is strengthened by the non-random pattern of significant interactions (e.g., significant interactions occurred at every age between intellectual impairment and the two categories of unemployment and education/training for self-rated health, see Table 4) and that the direction of effects was consistent across every single significant interaction. Finally, the temporal spacing of waves of data collection (every 4 years) precludes the analysis of the association between changes in employment state and health outcomes (an option that is possible in annual panel studies). As such, while the cohort is longitudinal, all our analyses are cross-sectional. Given this, we can only speculate on causal pathways.

## Conclusions

We undertook a series of cross sectional analyses of the association between employment status and health among British adults with and without intellectual impairments. The results provide substantive evidence to suggest that the nature of the well-established association between employment and better health is similar for British adults with and without intellectual impairments. The results do, however, indicate that the magnitude of effect sizes involved differed. Further research is needed to identify mechanisms that may underlie this difference.

## Abbreviations

IQ: Intelligence quotient
PRR: Prevalence rate ratio
